# IL-33/IL-31 Axis: A Potential Inflammatory Pathway

**DOI:** 10.1155/2018/3858032

**Published:** 2018-03-11

**Authors:** Eleonora Di Salvo, Elvira Ventura-Spagnolo, Marco Casciaro, Michele Navarra, Sebastiano Gangemi

**Affiliations:** ^1^IBIM-CNR Institute of Biomedicine and Molecular Immunology, National Research Council, 90100 Palermo, Italy; ^2^Legal Medicine Section, Department for Health Promotion and Mother-Child Care, University of Palermo, Via del Vespro 129, 90127 Palermo, Italy; ^3^School and Operative Unit of Allergy and Clinical Immunology, Policlinico “G. Martino”, Department of Clinical and Experimental Medicine, University of Messina, Messina, Italy; ^4^Department of Chemical, Biological, Pharmaceutical and Environmental Sciences, University of Messina, Messina, Italy

## Abstract

Cytokines play an important role in the regulation of the immune system (adaptive and innate). Given their importance in proinflammatory processes, cytokines have been used for understanding the pathogenesis and as biomarkers in many diseases. IL-31 and IL-33 are still considered novel cytokines. IL-31 controls signalling and regulates a huge amount of biological functions: it induces proinflammatory cytokines, regulates cell proliferation, and is involved also in tissue remodelling. On the other hand, IL-33 has been identified as an “alarmin” released from the epithelial cells and from different human tissues and organs after a damage following, that is, an inflammatory process. The aim of this literature review is to strengthen the hypothesis about an IL-31/IL-33 axis by evaluating the most recent studies linking these two cytokines. Literature data showed that, in many cases, IL-31 and IL-33 are linked to each other and that their expression is correlated with disease severity. The presence of one interleukin might stimulate the induction of the other, amplifying inflammation and the consequent detrimental processes. In a near future, influencing their balance could be helpful in modulating the first responses of the immune system in order to prevent the development of many inflammation-related diseases.

## 1. Introduction

Cytokines play an important role in the regulation of the immune system (adaptive and innate). Given their importance in proinflammatory processes, cytokines have been used for understanding the pathogenesis and as biomarkers in many diseases (i.e., atopic dermatitis, allergic inflammatory diseases, rheumatoid arthritis, asthma, skin diseases, and cancer) [1–7]. In these pathologies, it was demonstrated that cytokines act a main part in controlling the immune response [1].

During the last few years, the involvement of two novel interleukins, IL-31 and IL-33, emerged.

IL-31, a cytokine produced by CD4+ T helper cells, was identified for the first time in 2004 by Dillon et al. [8]. Its secretion depends on IL-4 [9, 10], but it is not only secreted by Th2 cells. Also other Th cell subsets that encounter IL-4 are able to release IL-31 [11]. This cytokine is a member of the gp130/IL-6 family, constituted by 4-helix (named A–D) bundle cytokines with 3 different receptor-binding sites [8, 12]. Most members of this family share the common chain of gp130 in their multiunit receptor complexes, except for IL-31, which uses IL-31RA and OSMR [8, 13]. Most gp130/IL-6 cytokines have a long-chain (20–30 amino acids): contrarily, interleukin-31 has two long helices (A and D, with 25–28 amino acid) and two short helices (B and C, with 10–16 amino acids) [14, 15].

IL-33 is a new cytokine belonging to the IL-1 family, which also includes IL-1*β* and IL-18, that appears to drive Th2 responses; in fact, interleukin-33 was demonstrated to induce the expression of Th2 cytokines. It is usually secreted by damaged tissues or sites of inflammation and acts as an alarmin in individuating damages in various inflammatory situations, including atopic dermatitis (AD) and skin diseases [16]. Therefore, IL-33 plays a critical role in the inflammation typical of allergic diseases mediated by the activation of basophils and eosinophils [17].

Furthermore, both IL-31 and IL-33 seem to activate and enhance the maturation of mast cells [16–18].

The aim of this study is to strengthen the hypothesis about the IL-31/IL-33 axis by evaluating the most recent studies linking these two cytokines.

## 2. Interleukin-31

**Figure 1:**
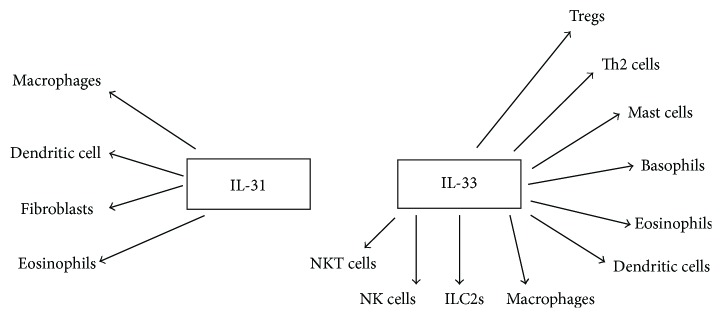
Immune cells activated by IL-31 and IL-33.

IL-31 is produced by the immune system cells, mainly by CD4+ T helper (Th2 cells) and mast cells, and it is known to exert its action on fibroblasts and eosinophils [8, 19, 20] (Figure 1). Interleukin-31 receptor, called IL-31R, takes its name from the ligand IL-31; it is a heterodimeric receptor, is ubiquitously expressed, and consists of 2 subunits, IL-31 receptor alpha (IL-31RA) and oncostatin-M receptor beta (OSMR), which are expressed on IL-31-activated monocytes. Four isoforms of IL-31RA were identified (IL-31RA-v1 to IL-31RA-v4) [11].

IL-31 controls the signalling that regulates a huge amount of biological functions: it induces proinflammatory cytokines, regulates cell proliferation, and is involved also in tissue remodelling [21]. Thus, IL-31 receptor complexes are primarily expressed in nonhematopoietic tissue, in the skin, and in the endothelium, suggesting that IL-31 has a number of functions in regulating these tissue responses [12, 16, 22].

IL-31 acts through three singling pathways: JAK/STAT pathway (Janus-activated kinase/signal transducer and activator of transcription), PI3K/AKT (phosphatidylinositol 3′-kinase/protein kinase) pathway, and MAPK (mitogen-activated protein kinase) pathway [12]. When IL-31 binds to its heterodimeric receptor composed of the IL-31RA/OSMR complex, it induces the phosphorylation of the JAK1/2, and subsequently it induces the phosphorylation of PI3K/AKT, and it activates STAT pathway [12, 23].

### 2.1. JAK and STAT Pathway

Transfection studies showed that the recruitment of those signalling pathways known to be common to IL-6 family cytokine receptors, included STAT1, STAT3, and STAT5, phosphoinositide-3-kinase, and ERK. These studies demonstrated the need of the cytoplasmic domain of IL-31R, as present in the full-length receptor subunit, to start signalling and determined tyrosine residues 652 and 721 (Tyr-652 and Tyr-721) to direct activation of STAT1/STAT5 and STAT1/STAT3, respectively [24]. The binding of IL-31 to its receptors activates powerful signalling pathways (i.e., the activation of Janus kinase JAK1 and JAK2 and the start of JAK-STAT pathway) [24, 25]. Phosphorylation and activation of kinases JAK1 and JAK2 triggers, lead in turn, to the phosphorylation of STAT3 and STAT5 and lesser of STAT1 [23].

It has been demonstrated that STAT signalling is negatively influenced through three ways: complete dephosphorylation of JAKs and of various protein tyrosine phosphates; inactivation of JAKs by the receptors of cytokine signalling proteins (SOCS); and inhibition of STATs and activation of PIAS protein (protein inhibitor of activated STAT).

### 2.2. PI3 and AKT Pathway

The same phosphorylation process is repeated in this pathway. In fact, when the IL-31RA and OSMR complexes are activated by IL-31, it starts an important tyrosine phosphorylation of PI3 (phosphoinositide-3-kinase), and so it is observed that PI3 is recruited and stimulates the PI3/AKT signalling pathway. In contrast to IL-31RA, which binds SH-2 (Src homology 2), the OSMR interacts with the adaptor protein Shc via the phosphorylated tyrosines on its intracellular domain [21, 26].

A malfunctioning of this pathway is involved in several human diseases such as cancer, diabetes, cardiovascular diseases, and neurological diseases [27, 28]. Chattopadhyay et al. showed that the control of cell proliferation occurs thanks to the activation of the STAT receptors, activated in turn by cytokines; the cytokines belonging to the IL-6 family have gained particular attention in order to explain their role in tissue damage and repair and also for their potential relevance in supporting tumorigenesis [24].

### 2.3. MAPK Pathway

In this pathway, there is an extracellular signalling that activates a conformational change of GRB-2 mitogen-activated protein kinases (MAPKs). The activation of MAPKs is fundamental for the development of IL-31-induced bronchial inflammation. Elevated plasmatic levels of IL-31, in patients affected by allergic asthma, could induce JAK to phosphorylate Shc leading to the activation of the ERK1/2 pathway [12, 29].

## 3. Interleukin-33

IL-33 has been identified as an “alarmin”; it is released from the epithelial cells and from different human tissues and organs. IL-33 could enhance T cell response and also causes the maturation and activation of human mast cells (Figure 1) [17, 30]. Recently, several authors proposed that IL-33 may have a dual function, acting extracellularly as an IL-1 family cytokine and intracellularly as a nuclear factor regulating gene expression [31–35]. Infectious agents and irritants can stimulate the innate immune system to produce IL-33. This interleukin exerts its action, on the surface of the above cited cells, by the receptor T1/ST2 (ST2) [36]. This receptor is also involved in the secretion of proinflammatory factors, such as leukotrienes, IL-6, and TNF-alpha. These factors can cause vasodilatation with a consequent increased permeability of the microvessels and the subsequent infiltration of inflammatory cells [37]. Moreover, IL-33 has powerful effects on many cell types such as ILC2s (type 2 innate lymphoid cells), mast cells, eosinophils, and Th2 lymphocytes. In particular, IL-33 could play a major role in ILC2 recruitment; in fact, recent studies have demonstrated that IL-33 activates ILC2s, which induce type 2 lung inflammation. According to these data, a key role of IL-33 in the activation of ILC2s in humans emerged [38].

### 3.1. IL-33/ST2 Signalling

At first, a split by caspase-1 that leads to the maturation and the activation of the interleukin (from pro-IL-33 to IL-33) occurs.

ST2 is a member of the toll-like/IL-1-receptor superfamily. ST2 is the only receptor for IL-33, but it has four splice isoforms from a single transcript based on the promoter involved. It owns an extracellular domain, which binds interleukin-33 helped by the IL-1 receptor accessory protein (IL-1RAP), constituted by a transmembrane domain, and by an intercellular domain called a toll/interleukin-1 receptor (TIR) [39].

The latter domain, consisting of ~160 amino acids, is made by a central five-stranded sheet rounded by five helices placed on the cytosolic end of the protein. Moreover, the toll receptor subfamily is constituted by extracellular leucine-rich repeat motifs, which has its representation in the toll-like receptors TLR from 1 to 12; these receptors serve as gateways to proinflammatory signalling pathways [40].

Once the IL-33, ST2, and IL-1RAP are linked, the heterodimer leads to the dimerization of the TIR domain, necessary for the activation of many signalling pathways.

Thus, the adaptor protein, named MyD88, activated in turn by the heterodimeric complex, activate downstream the following: (1) IARK-1, IARK-4, and mitogen-activated protein kinase (MAPK) kinases, through the TNF receptor-associated factor 6 (TRAF6) signalling, which in turn activates activator protein 1 (AP-1) through c-Jun N-terminal kinases (JNKs). TRAF6 also activates the inhibitor of nuclear factor-*κ*B (NF-*κ*B) kinase (IKK) complex, leading to a downstream release of active NF-*κ* from the complex; this pathway activates nuclear transcript signals fundamental for the production of other inflammatory cytokines. (2) Jun kinase (JNK) and (3) extracellular signal-regulated kinase (ERK)1/2 following receptor ligation promote an activation of IRF1 that inhibits Foxp3 and GATA3 expressions [1].

Furthermore, MyD88 also promotes Treg function and expansion enhancing TGF-*β*1-mediated differentiation though a p38-dependent mechanism [41].

Recently, a study demonstrated that IL-33 binds a receptor complex constituted by ST2L and IL-1RAcP (receptors accessory protein of IL-1). In addition, the affinity of IL-33 for ST2L is ameliorated by the presence of IL-1RAcP [40].

All these molecular mechanisms suggest a potential role of IL-33 in many pathologies, especially in the inflammatory ones, and also in the equilibrium of the immune response (Th2-associated). Thus IL-33 seems to be closely associated with allergic inflammatory diseases, including atopic dermatitis and asthma [42].

## 4. IL-31 and IL-33 in Diseases

**Figure 2:**
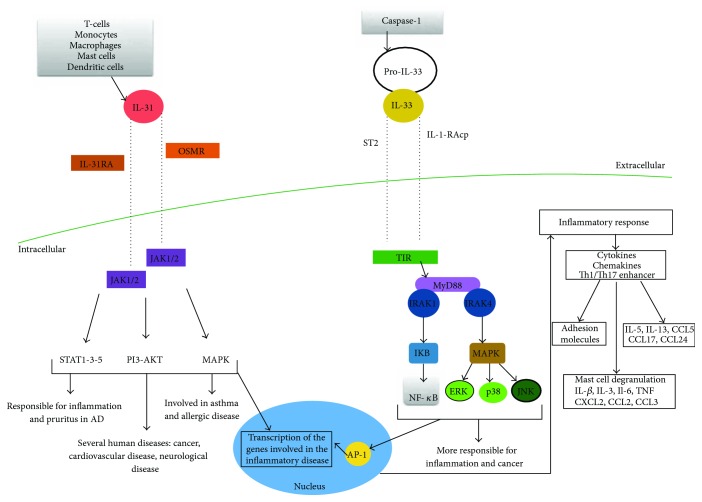
Visual description of the IL-31 and IL-33 cascades leading to the activation of the genes and the proteins involved in the development of inflammatory disorders. IL: interleukin; OSMR: oncostatin-M-specific receptor beta; IL-31-RA: interleukin-31 receptor A; JAK: Janus kinase; STAT: signal transducers and activators of transcription; PI3: phosphoinositide-3-kinase; AKT: protein kinase B; MAPK: mitogen-activated protein kinase; AD: atopic dermatitis; ST2: soluble receptor; IL-1RAcP: IL-1R accessory protein; TIR: toll/interleukin-1 receptor; MyD88: myeloid differentiation primary response protein; IRAK: interleukin-1 receptor-associated kinase; IKB: I kappa B; NF-*κ*B: nuclear factor- (NF-) *κ*B; ERK: extracellular signal-regulated kinase; JNK: Jun N-terminal kinases; Th: T helper; CCL: chemokine (C-C motif) ligand; TNF: tumor necrosis factor.

Many cytokine studies were conducted on skin allergic diseases, due to their suitability as inflammation models. In these conditions, it was demonstrated that T cell-mediated inflammation has a main role in the development of several skin diseases such as atopic dermatitis (AD), allergic contact dermatitis (ACD), and psoriasis; IL-31 and IL-33, as novel cytokines, were firstly investigated in the above-cited pathologies [12]. In Figure 2, there is a summary of IL-31 and IL-33 cascades leading to the activation of the genes and the proteins involved in the development of inflammatory disorders.

### 4.1. Atopic Dermatitis (AD)

IL-31 was shown to be upregulated in patients with atopic dermatitis, but it has not yet been clarified whether it is the allergen or a secondary factor that induces the expression of this cytokine; literature results showed that IL-31 activation could occur directly by allergen stimulation. In particular, it was supposed that cells producing IL-31 were allergen-specific memory T cells [11]. Both Th1 and Th2 cytokines could take part in the IL-31 pathway. Thus, IL-31 was regarded as a novel player in type 2 inflammation. This theory was supported by the discovery that Th2 cells are one of the main producers of IL-31 [10]. In fact, some studies demonstrated a positive correlation between IL-31 and AD severity [43]. These data suggest an important role of IL-31 in the regulation of AD.

Instead, the role of IL-33 in atopic dermatitis has not been clarified; it seems that IL-33 in skin can convert an innocuous antigen exposure into an allergen sensitization leading to a skin reaction [44].

It is possible that a faulty innate or adaptive immune response can induce a skin disease, which could cause secondarily an increased Th2 response (with an upregulated IL-31 and IL-33 production). This sequence could be the basis for the worsening of skin diseases, inducing pruritus, wounding, and worse bacterial infection [17].

A recent study demonstrated that a p53 family member p63 (ΔNp63) has a fundamental function in driving, developing, and differentiating the keratinocyte activation in AD [45]. ΔNp63 could be a key upstream mediator in the development of AD, where, along with this factor, there are elevated levels of IL-31 and IL-33. The elevated IL-31, IL-31RA, OSMR, and IL-33 are direct targets of ΔNp63, contributing to specific symptoms such as itching, skin lesions, and localized irritation/inflammation typical of AD [45].

### 4.2. Inflammatory Diseases

A great amount of data demonstrated that IL-31/IL-31R interaction may play a central role in limiting the Th2-mediated inflammatory response [46, 47]. From other reports, it emerged that IL-31 has often a positive correlation with skin inflammation [48]. Thus, it was reported that the transcription factor STAT1, interferon-*γ*-dependent, is fundamental for IL-31RA expression. Once expressed, the receptor becomes responsive to IL-31 and participates in the secretion of a series of cytokines and chemokines involved in proinflammatory processes [49]. However, a 2010 study on IL-31RA-deficient mice suggested that IL-31 may limit Th-2 mediated inflammation [50].

IL-31 could take part in a positive feedback loop in the progression of skin inflammation because it can induce the secretion of specific mediators, which can in turn promote inflammation by activating dendritic cells (DCs) [49].

In addition, IL-33 is considered a modulator of inflammation, pushing the balance in the direction of a CD4+ T helper-cell type 2-mediated immune response [40].

Several studies demonstrating a role of IL-33/ST2 in diseases associated with a Th2 response (i.e., asthma) remarked that ST2L is a cell surface marker in addition to its function as an effector molecule in the regulation of Th2 cell pathway [40, 51, 52].

### 4.3. Asthma

Some researches demonstrated that the expression of IL-31 [11] and IL-31R [53] was increased in allergic diseases, especially in asthma. On the other hand, IL-33 was found to be involved in the progression of many chronic inflammatory and autoimmune diseases. It promotes both the innate immune response and also the adaptive immune response. In fact, in different studies, it was reported that there are increased expressions of IL-33 and ST2 in lung tissue of asthmatic and allergic airways and that ST2 plays a crucial role in antigen-induced airway inflammation [40]. IL-33 could induce bronchial asthma because it is increased during the production of inflammatory cytokines by Th2 cells [54].

### 4.4. Autoimmune Diseases

Guerrero-García et al. demonstrated that the soluble form of CD40L was an excellent marker for inflammatory and autoimmune diseases. Furthermore, they found in serum the positive and close correlation between IL-31 and autoimmune diseases like multiple sclerosis [55].

Moreover, the role of IL-33/ST2 in a vast array of autoimmune diseases emerged, because of the detection, in patients' serum, of elevated ST2 levels [40].

#### 4.4.1. Rheumatoid Arthritis (RA)

Rheumatoid arthritis (RA) is an autoimmune disease that is characterized by inflammatory cells joint infiltration, leading to cartilage and bone destruction. Dysregulated macrophage responses were shown to contribute to many autoimmune diseases pathogenesis by the production of proinflammatory cytokines such as IL-33, ST2, and IL-1*β*, as well as proinflammatory chemokine (MCP-1). The depletion of macrophages alleviates the symptoms and the severity of collagen-induced arthritis (CIA). In fact, the concentration of macrophages is markedly elevated in the inflamed synovial tissues of RA patients and positively correlated with disease pathogenesis and progression.

Chen et al., in their study, determined the direct contribution and the action mechanism of IL-10 in autoimmune inflammation by using IL-10-deficient mice. They described for the first time that IL-33 expression is positively correlated with IL-10 level in patients with active RA. Importantly, it was found that macrophages are responsive to IL-10-STAT3 pathway in controlling IL-33 production. Furthermore, IL-33 induced the production of proinflammatory cytokines by macrophages [56].

#### 4.4.2. Systemic Lupus Erythematosus

Systemic lupus erythematosus (SLE) deserves its own paragraph as it is one of the most common and well-studied autoimmune diseases and as it is a systemic disease that involves various organs. Recently, it was demonstrated that a disorder of cytokine balance appears to act an important role in the pathogenesis of SLE. It was also suggested that there is a functional imbalance of Th1 and Th2 cells. In SLE patients, in fact, Th1 response is decreased, whereas the Th2 one is elevated. This suggests that IL-31 could play an important role in SLE patients. A recent study demonstrated that the IL-31 gene may contribute to an inherited predisposition to SLE [57].

Moreover, SLE patients resulted in having noticeably higher serum IL-33 levels [52, 58]. In addition, IL-33 gene polymorphisms may increase the susceptibility to SLE and could be a biomarker for disease activity in SLE [59].

### 4.5. Cardiovascular Diseases

As far as cardiovascular diseases are concerned, the involvement of IL-31 has been studied in Kawasaki's disease. In particular, a study demonstrated how IL-31 expression was related to Kawasaki's disease, and thus, its higher expression could be considered as a promoter of coronary lesions [60]. On the other hand, serum levels of ST2 in patients affected by heart failure and myocardial infarction were found to be elevated, and the increase of ST2 levels was proposed as a biomarker in forecasting the risk for a cardiac transplantation and the risk of heart failure [40].

## 5. Discussion

As shown above, IL-33 is a multifunctional cytokine acting a main role in a variety of biological processes, such as in the immune response, tissue homeostasis equilibrium, growth, and repair; these researches suggest that IL-33 is involved in the pathogenesis of various human pathologies. In particular, IL-33 is also known as being an “alarmin” because its serum level increases as a consequence of necrosis processes and induces the augmentation of inflammatory cytokines. According to literature, IL-33 has main target tissues like airways and skin. Moreover, many diseases associated with inflammatory detrimental effects seem to be linked to this interleukin. The mechanisms involved are sophisticated and include different kinds of cells. It is plausible to consider IL-33 as a potential routine biomarker for diseases associated with a cell damage like asthma, COPD, AD, RA, and heart failure [61].

On the same path, results obtained during the last years suggested an analogue potential for IL-31, not only due to its role in the pathogenesis of some diseases (AD, prurigo, chronic urticaria, psoriasis, vasculitis, connective tissue diseases, asthma) but also because of the hypothesis speculated by Wong [26] firstly and then extended by our group [16, 18, 37, 48]. In fact, the IL-33 secreted as consequence of cell damage promotes the IL-4-dependent release of IL-31 by CD4+ Th cells [62]. Although recent data demonstrated that IL-31 signalling is required to limit Th2 cytokine-driven inflammation in the lung [50, 63], other studies showed also that, in many cases, both IL-31 and IL-33 were correlated with the intensity of the symptoms and with severity of the signs. In fact often AD, pruritus, and wheals were associated with the level of the interleukins and in chronic inflammatory lung diseases as the higher the levels were, the more the relapse; since a correlation exists, IL-31 and IL-33 dosage could be used as a disease severity monitor and as a biomarker of therapy efficacy.

In addition, previous studies on serum showed that levels of IL-31 and IL-33 were very high, suggesting a close correlation between these two interleukins in many pathologies.

A variety of cytokines plays a fundamental role in inflammatory processes; they can evolve into unstoppable and detrimental processes such as necrosis. The “alarmin” IL-33 increases its expression after cell death, and, probably, it results in the induction of other cytokines including IL-31. What emerged from the researches cited above is a tight correlation between serum and tissue levels of IL-31 and IL-33 together with the involvement of their imbalance in inflammatory disorders. These data make us speculate the existence of a correlation axis between these two novel cytokines. In particular, Vocca et al. [64] showed that the IL-33/ST2 axis might be involved in the progression of the various pathologies, influencing the generation of Th17 producing IL-31; they found very high serum levels of IL-31 and IL-33 in many inflammatory and autoimmune diseases, especially in lung illnesses [42, 64]. These data suggest that the activation of the IL-33/ST2 axis, which can be considered also as a biomarker of both Th2/IL-31 and Th17 immune response, might represent a link between the respiratory system and immune system. For this reason, it can be useful for the diagnosis, the control, and the evaluation of the activity and progression of many inflammatory diseases [16, 18, 37, 64, 65]. As the number of STAT proteins is lower than the number of cytokines, it is therefore probable that different cytokines can induce the activation of a single STAT. For example, it has been demonstrated that the receptors of the two cytokines, IL-31RA and ST2, are both expressed on dermal fibroblasts; it was hypothesized that IL-31 and IL-33 could synergistically stimulate chemokines in AD, supporting the involvement and the connection of both IL-31 and IL-33 in the various diseases, especially in AD [16]. Nygaard et al. observed an intimate link between the immunomodulatory cytokines TSLP, IL-33, and IL-31 and discovered a negative correlation between IL-33 and sST2 and a positive one between IL-33 and IL-31 [65].

## 6. Conclusion

Thanks to these data, recent studies have focused their attention on regulating the balance of these cytokines for the treatment of inflammatory disorders; future studies should focus on predicting the possible benefit of therapeutic and preventive approaches on these two novel cytokines and on their receptors [66–72]. An increasing knowledge in this field is giving us a new perspective for the development of new therapies with maybe less side effects than the conventional ones have [73]. Targeted biological therapies are the most upcoming ones for the treatment of allergic diseases (i.e., monoclonal antibodies and fusion proteins against cytokines or their receptors). IL-31, considered to be responsible for the development of pruritus in AD, was one of the first therapeutic targets. Recently, in a clinical trial, nemolizumab (anti-IL-31RA) administration confirmed its safety and efficacy on the reduction of pruritus [74].

However, creating bispecific antibodies targeting more than one element should be a future approach; that is, a bispecific antibody with affinity for both IL-31 and IL-33 receptors could be developed [75].

Probably we are close enough in using IL-31 and IL-33 as new diagnostic, prognostic, and follow-up biomarkers; they could also be useful in monitoring treatment responses. New studies could focus on the combination of the two interleukins, IL-31 and IL-33, as disease biomarkers; the combination of data could let us better understand their relationships and identify a possible benefit of a combined therapeutic approach. Modifying their balance could be helpful in modulating the first responses of the immune system fundamental for the development of the above-described diseases.
